# Cognitive considerations for adults with sickle cell disease completing the brief pain inventory

**DOI:** 10.1097/PR9.0000000000001189

**Published:** 2024-12-09

**Authors:** Julia A. O'Brien, Jermon A. Drake, Donald J. Bearden, Kim E. Ono, Soumitri Sil, Lindsey L. Cohen, Alana Karras, Enrico M. Novelli, Charles R. Jonassaint

**Affiliations:** a School of Nursing, University of Pittsburgh, Pittsburgh, PA, USA; b Department of Psychology, University of Pittsburgh, Pittsburgh, PA, USA; c Emory University School of Medicine, Atlanta, GA, USA; d Children's Healthcare of Atlanta, Atlanta, GA, USA; e Department of Psychology, Georgia State University, Atlanta, GA, USA; f School of Medicine, University of Pittsburgh, Pittsburgh, PA, USA; g School of Medicine, Department of Medicine, Division of Hematology/Oncology, University of Pittsburgh, Pittsburgh, PA, USA; h Center for Research on Health Care, University of Pittsburgh, Pittsburgh, PA, USA; i Center for Behavioral Health and Smart Technology, University of Pittsburgh, Pittsburgh, PA, USA

**Keywords:** Sickle cell disease, Pain, Measurement, Sickle cell anemia

## Abstract

**Introduction:**

Accurate assessment of pain severity is important for caring for patients with sickle cell disease (SCD). The Brief Pain Inventory was developed to address limitations of previous pain-rating metrics and is available in a short form (BPI-SF). However, the BPI-SF is a self-report scale dependent on patient comprehension and interpretation of items.

**Objective:**

To examine patterns in how patients completed the BPI-SF and determine whether incorrectly completing the BPI-SF was related to cognitive functioning or education.

**Methods:**

A secondary analysis was completed using data from a study examining brain aging and cognitive impairment in SCD. T-tests were performed to examine whether neurocognitive function (immediate and delayed memory, visuospatial skills, attention, and language), word reading, and years of education differed based on correct BPI-SF completion.

**Results:**

The sample (n = 71) was 43.7% male, 98.6% African American or mixed race. Of that, 53.5% had sickle cell anemia, and the mean years of education was 13.6. Overall, 21.1% of participants (n = 15) incorrectly completed the BPI-SF pain severity items, and 57.7% completed the body map item incorrectly. Those who completed the severity items incorrectly had statistically significant differences in education. Group differences in neurocognitive function were no longer significant after familywise error rates were controlled for. Literacy was not associated with error rates.

**Conclusion:**

Education level may influence patients' ability to correctly complete the BPI-SF. Findings suggest that careful consideration is warranted for use of the BPI in patients with SCD. Recommended revisions to the BPI include simplifying the language, shortening sentence length, and clearly specifying the timeframes.

## Abstract 2

Supplemental Digital Content is Available in the Text.

Inconsistent pain severity ratings are associated with lower education in adults with sickle cell disease. Simpler self-report instruments may aid in accurately measuring pain.

## 1. Background

Pain is the most common symptom associated with sickle cell disease (SCD),^22^ a genetic blood disorder that primarily affects individuals from world regions affected by malaria and their descendants.^38,53^ Fifty percent of adults with SCD have chronic pain, and of those, 30% report being in pain every day.^59^ Due to the sickling of red blood cells under hypoxic conditions, clotting of blood vessels, and tissue reperfusion injuries, patients with SCD experience acute painful events throughout the body known as vaso-occlusive events (VOEs).^3^ Chronic pain may develop as early as infancy and continue into adulthood.^45^ Because patients with SCD experience both acute and chronic pain frequently, it is particularly challenging for patients with SCD to communicate their pain experience accurately and comprehensively.^17^

Accurate and comprehensive pain assessment is a prerequisite to successful pain treatment, and it must include multiple pain dimensions such as physiologic, sensory, affective, cognitive, behavioral, and sociocultural aspects of pain, as well as the impact of pain on daily life.^22^ Commonly used^22^ unidimensional scales such as the Numeric Rating Scale (NRS),^32,64^ the Visual Analogue Scale (VAS),^32^ and the Bieri Faces Pain Scale^8^ focus primarily on pain severity at the time of health care encounters, thus are limited in their ability to characterize the complex pain of patients with SCD. Comprehensive pain assessment tools are required.

The brief pain inventory (BPI) is a multidimensional tool used for assessing pain.^16^ The BPI is self-administered and assesses pain severity and impact of pain on daily functioning.^16,39^ The BPI was initially designed for patients with cancer,^5,16,28,42,58^ but it is used to measure chronic and acute noncancerous pain in epidemiological studies and clinical trials^4,11,29,39,57^ and is recommended for use with SCD.^22^ Collins, Renedo, and Marston propose that patients asked to describe their pain using numerical scales undertake a series of tasks, including interpreting the scope of the questions (eg, time, the type of pain, etc), interpreting what the numbers in the scale represent, translating their pain experience into a number, and then if needed, adjusting the rating to achieve their goals in the health care interaction.^17^ But pain perception is highly subjective,^22^ and SCD pain is uniquely complex because it may be acute, chronic, nociceptive, neuropathic, or a combination of these,^3^ making it challenging for patients to communicate their experiences via clinical pain scales^17^ like the BPI.

Our group previously conducted a retrospective review of the BPI short form (BPI-SF) completed by adults with SCD during a routine clinic visit to determine if it was being completed accurately.^15,25^ The study found that 51% of participants erred on pain severity items (eg, rating their least amount of pain as higher than their highest level of pain experienced within the timeframe, etc), 80% erred on the body image item, and 45% had one or more missing items. This was likely a result of misinterpreting or not thoroughly reading written instructions. However, these errors may represent deficits in cognitive functioning that are commonly observed among adults with SCD.^43,50,63^

Neurocognitive problems and limited education may influence the ability of patients with SCD to complete the BPI, which requires integration of multiple cognitive abilities.^62^ Sickle cell disease increases the risk for neurocognitive deficits and dementia due to disease-related complications such as overt and silent strokes,^24,36^ associated hypoxia,^34,54^ and cerebrovascular stenosis,^18^ as well as other inflammatory and coagulative processes that may contribute to cognitive decline. Strokes in patients with SCD have been associated with deficits in multiple cognitive abilities, including executive function,^1,12,63^ memory,^43^ language,^23^ attention,^1,12,21^ and verbal comprehension,^50^ but even pediatric patients with no evidence of silent stroke are more likely to have neurocognitive deficits compared to healthy peers.^56^ Relatedly, SCD is associated with poor academic attainment due to VOEs, neurological events, and frequent medical visits that result in school absenteeism beginning in early life.^33^ This study examines BPI-SF response patterns in patients with SCD and cognitive correlates that may affect reading ability and interpretation of questions.

## 2. Methods

Data were collected as part of a longitudinal cohort study examining brain aging and cognitive impairment in SCD (ClinicalTrials.gov Identifier: NCT02946905). The parent study included a cohort of 113 participants with SCD^50^ who underwent a battery of neurocognitive tests, surveys of pain and mood, magnetic resonance imaging and blood collection, and an age-, race-, and a sex-matched group of 84 participants with non-SCD. The participants with SCD completed a clinical visit at baseline (T1) and at 3 years (T2). Participants were recruited from the UPMC Adult Sickle Cell Program outpatient clinic by the PI and study staff. Informed consent was obtained prior to administering questionnaires or accessing patient records for clinical or pain data. Demographic data, neurocognitive tests, and the BPI were collected during scheduled study visits. If participants did not have adequate time to complete all study questionnaires, they continued the surveys during their next clinical encounter. Participants who were unable to fully complete cognitive tests due to impairment were assigned a score of -1 the lowest score in the sample so that highly impaired individuals were not excluded from analyses.

### 2.1. Ethical oversight

The research activities were approved by the IRB at the University of Pittsburgh (STUDY19110198).

### 2.2. Measures

Measures used in these secondary analyses include a patient demographic questionnaire and stroke history, the BPI-SF, the Cumulative Illness Rating Scale—Geriatric (CIRS-G), the Repeatable Battery for the Assessment of Neuropsychological Status (RBANS),^52^ Hopkins Verbal Learning Test–Revised (HVLT-R),^6,10^ and Wide Range Achievement Test 4 (WRAT-4) Reading Subtest^37^ to assess participant reading level. The questionnaires were administered to patients receiving routine care at a comprehensive sickle cell clinic via paper and pencil and scored by trained reviewers.

#### 2.2.1. The brief pain inventory short form

The BPI-SF is a 9-item scale that measures pain severity and impact on function.^15,16^ Item 1 is a yes/no question asking whether participants have pain on the day of the test. Item 2 is a body map on which participants shade the area they feel pain and place an X where they feel the most pain. Items 3, 4, 5, and 6 ask participants to rate their worst, least, average, and current pain in the past 24 hours on a 0 to 10 Likert scale, 0 representing “no pain” and 10 representing “the worst pain imaginable.” Item 7 asks patients to list their current pain medications, and item 8 asks how much relief pain treatments provided. Item 9 measures pain interference, in which participants rate the degree to which pain has interfered with their daily life within the previous 24 hours. The interference items also use a 0 to 10 Likert scale, 0 representing “not at all” and 10 representing “completely interferes.” Items 3 to 6 can be summed for a total pain severity score ranging from 0 to 40, with higher scores indicating greater severity. The BPI-SF and scoring guide can be viewed and licensed from MD Anderson Cancer Center.^44^

#### 2.2.2. Cumulative illness rating scale—geriatric

The CIRS-G is a measure of chronic medical illness burden that contains 14 organ-system categories (heart, vascular, neurological, etc.).^46^ After chart review, these categories are rated from 0 to 4, representing “no problems” to “severe impairment.” These data are then used to generate the total number of categories endorsed, a total score ranging from 0 to 56, a severity score consisting of the ratio of total score divided by the number of endorsed organ-specific categories, and the number of categories at level 3 and 4 for a given patient. We examined differences in the total and severity scores for these patients with SCD.

#### 2.2.3. Repeatable battery for the assessment of neuropsychological status

The RBANS is a well-validated, reliable, standardized neuropsychological screening tool^51,52^ that has been used previously to evaluate cognitive impairment among adults with SCD.^41,50^ It contains 12 subtests that examine immediate memory (list learning and story memory), delayed memory (list recognition, recall, story recall, and figure recall), visuospatial/constructional skills (figure copy and line orientation), language (picture naming and semantic fluency), and attention (digit span and coding), yielding 5 index scores ranging from 40 to 154 and a total score ranging from 40 to 160 that represents overall cognitive status. These scores can be transformed into age-corrected normative standard scores (M = 100, SD = 15).^52^

#### 2.2.4. Hopkins verbal learning test–revised

The HVLT-R is a well-validated and reliable measure of rote verbal learning and memory^6,10^ that has been used to evaluate cognitive abilities in adults with SCD.^50^ The HVLT-R consists of a list of 12 nouns with 4 words drawn from each of 3 semantic categories that are presented to participants over 3 consecutive trials, immediately after which they are required to spontaneously recall as many words as possible. For the delayed recognition task, they are asked to recall the words again 20 to 25 minutes later. The 3 learning trials are summed to create the total score with a range of 0 to 36, and the delayed recall score (0–12) calculated as the number of words recalled during the delayed recognition task.^26^ Raw HVLT-R scores are converted to age-corrected normative standard scores that generally range from 20 to 80, with M = 50, SD = 10.

#### 2.2.5. Wide range achievement test 4 Word Reading Subtest

Performance on standardized word reading tests is frequently used as a proxy for reading comprehension level.^57^ The WRAT-4 Word Reading Subtest is a 70-item oral reading test that measures an individual's ability to recognize letters and words.^37^ During administration, participants are asked to name letters and pronounce words out of context, with decreased familiarity and increased phonological complexity as the test advances. Each correctly identified letter or pronounced word is worth 1 point; raw scores are converted to age-corrected normative standard scores with a mean = 100, SD = 15, range 55 to 145.^37^

### 2.3. Study cohort and procedures

For the current study, the cohort was limited to individuals with SCD who completed the BPI-SF at either the initial (T1) or second visit (T2) of the parent study. If participants completed the BPI-SF at more than one time point, the most complete BPI-SF form was used. Of the parent study's recruited 113 subjects, 71 completed BPI-SF forms.

All data were entered into REDCap^30,31^ and exported into Statistical Package for the Social Sciences (SPSS Statistics 28)^19^ for analyses. Completed BPI-SFs were examined for the following mistakes: (1) stating they had no pain today (item 1) but subsequently reporting pain severity greater than zero “right now” (item 6); (2) conflicting data entered for numeric scales of worst (item 3), least (item 4), and current pain (item 6); and (3) missing at least one pain severity item. See Figure 1 in the supplementary materials, http://links.lww.com/PR9/A246 for further information.

In addition, we found the validity of responses to items 2 and 5 difficult to assess. Item 2 provides instructions for completing the body map that are open to interpretation and result in considerable variability in answers (eg, patients are asked to mark an X where they have the most pain; some participants put multiple Xs on the body map, whereas some put no Xs at all). Item 5 asks about “average” pain but does not explicitly state a timeframe, which may affect ratings (eg, their “average” pain may be rated higher than their “worst” pain). Thus, we chose to consider these data as *potentially incorrect* and reported them separately. Items 7–9 were not explored in the current study, because other than missingness there are no concerns about the validity of the participants' responses.

### 2.4. Data analyses

Descriptive statistics were used to evaluate the frequency of errors among the BPI-SF pain severity entries. Independent *t*-tests were used to compare neurocognitive function, reading ability, and education level using the RBANS, HVLT-R, WRAT-4 Word Reading Subtest, and years of education among those who did/did not complete the BPI-SF correctly. To account for multiple comparisons, *P*-values were adjusted using the Benjamini–Hochberg methods and a false-discovery rate of 0.05. All statistical assumptions were assessed and met; all variables were normally distributed and had homogeneity of variance. Possible outliers were assessed via boxplots, and then *t*-tests were compared with and without these cases, but we found no differences in the analyses.

## 3. Results

Demographic data for this study are presented in Table 1. Mean age of the sample was 37.03 (SD = 12.05, range = 20–70). Males made up 43.7% (n = 31) of our sample and females 56.3% (n = 40). Participants identified as African American or mixed race (98.6%) or White/Caucasian (1.4%). Sickle cell disease type included 53.5% (n = 38) with HbSS, 36.6% with HbSC, 8.5% with SB + Thal, and 1.4% with SB0Thal. Mean years of education was 13.6 (SD = 2.04, range = 8–18 (n = 53)). We found that both groups had similar rates of stroke and CIRS-G scores, indicating similar levels of illness burden.

**Table 1:** Demographics & descriptive statistics of the sample (n = 71).

Variable - n (%)	Correct BPI (n = 56)	Incorrect BPI (n = 15)	Total (n = 71)	*P*
Sex				0.723
Female	33 (58.9%)	7 (46.7%)	40 (56.3%)	
Male	23 (41.1%)	8 (53.3%)	31 (43.7%)	
Race				>0.999
African American (AA) or mixed race AA	55 (98.2%)	15 (100%)	70 (98.6%)	
White	1 (1.8%)	0 (0%)	1 (1.4%)	
SCD Genotype				0.657
Severe Genotypes	30 (53.6%)	9 (60%)	39 (54.2%)	
HgbSS	29 (51.8%)	9 (60%)	38 (53.5%)	
SB^0^Thal	1 (1.8%)	0 (0%)	1 (1.4%)	
Milder Genotypes	26 (46.4%)	6 (40%)	32 (45.1%)	
HgbSC	22 (39.3%)	4 (26.8%)	26 (36.6%)	
SB^+^Thal	4 (7.1%)	2 (13.3%)	6 (8.5%)	
Employment (n = 65)	n = 50	n = 15	n = 65	0.881
Disabled	6 (12.0%)	3 (20%)	9 (13.8%)	
Unemployed	11 (22.0%)	2 (13.3%)	13 (20.0%)	
Part time	3 (6.0%)	2 (13.3%)	5 (7.7%)	
Full time	10 (20%)	2 (13.3%)	12 (18.5%)	
Student	6 (12.0%)	2 (13.3%)	8 (12.3%)	
Retired	2 (4.0%)	2 (13.3%)	2 (3.1%)	
Other	12 (24.0%)	4 (26.7%)	16 (24.6%)	
Marital status (n = 67)	n = 53	n = 14	n = 67	0.632
Cohabiting	1 (1.9%)	0 (0%)	1 (1.5%)	
Divorced	4 (7.5%)	0 (0%)	4 (6.0%)	
Married	7 (13.2%)	3 (21.4%)	10 (14.9%)	
Never married	41 (77.4%)	11 78.5%)	52 (77.6%)	
Stroke history	n = 51	n = 13	n = 64	0.672
No	43 (84.3%)	12 (92.3%)	51 (79.7%)	
Yes	8 (15.7%)	1 (7.7%)	13 (20.3%)	
Variable mean (SD)				
Age (y)	36.70 (12.74)	38.27 (9.3)	37.03 (12.05)	0.657
Education (y)	n = 51 14.02 (1.84)	n = 12 12.17 (2.13)	n = 53 13.6 (2.04)	0.004*
CIRS-G	n = 40	n = 12	n = 52	
Total score	5.05 (3.22)	4.00 (2.13)	4.81 (3.02)	0.295
Severity index	1.43 (0.39)	1.60 (0.43)	1.47 (0.40)	0.205
Brief pain inventory*	n = 38	n = 14	n = 52	
Item 3	5.66 (2.85)	6.00 (2.45)	5.75 (2.73)	0.693
Item 4	2.50 (2.11)	5.00 (2.90)	3.17 (2.58)	0.001*
Item 5	4.50 (2.44)	5.79 (2.23)	4.85 (2.43)	0.091
Item 6	3.82 (2.69)	4.07 (1.98)	3.88 (2.5)	0.747
Total pain severity	16.47 (9.14)	20.86 (7.79)	17.65 (8.94)	0.118

A total of 71 BPI forms were completed. However, some participants may have stopped at item 1 if they answered “no” (Have you had any pain in the past 24 hours) and not completed pain severity measures. While presumably they would have rated items 3–6 as 0 (no pain), these items were left blank; therefore, these subjects are not included in this section of the demographic table.

* *P* < 0.05.

BPI, brief pain inventory; CIRS-G, Cumulative Illness Rating Scale—Geriatric; HgbSC, hemoglobin SC disease; HgbSS, sickle cell anemia; N, sample size; SB + Thal, Sickle beta^+^-thalassemia; SB0Thal, Sickle beta^0^ -thalassemia; SD, standard deviation.

### 3.1. Descriptive statistics

BPI-SF forms were coded for inconsistencies and descriptive data were examined. Pooled BPI-SF assessments of 21.1% (n = 15) participants contained inconsistencies as follows: 2.8% (n = 2) of participants indicated no pain today (item 1) but reported above-zero pain severity currently (item 6), 15.5% (n = 11) had conflicting scores on the numeric pain severity items 3, 4, and 6 (eg, lowest pain in the past 24 hours was rated as more severe than the highest pain in the past 24 hours, or other mismatches in the timing and severity of reported pain), 2.8% (2) reported the same level of pain severity across all Likert scale items (possibly indicative that patients did not fully understand the question), and 1.4% (1) had missing data. For item 5, 13 participants (18.3%) reported an average score outside the range for least and worst pain, but because this question asked for “average pain,” rather than “average pain in the past 24 hours,” we did not count this response as incorrect.

Item 2 (the body map) was examined separately for potentially incorrect answers. We found that 41 participants (57.7%) completed the body map item in ways that conflicted with instructions or were difficult to interpret, 9 participants (12.7%) completed the item incorrectly (eg, put Xs outside of the body map, used a mark other than X, etc), and 19 (26.8%) did not complete the body map because they indicated that they had no pain in item 1. Because the item 2 instructions may be open to interpretation and many respondents left the item blank, we examined potential differences in cognitive function separately from the pain severity items and included this in the supplementary materials, http://links.lww.com/PR9/A246.

### 3.2. Comparisons of cognitive function between error groups

Independent *t*-tests were used to examine differences in performance on the RBANs, HVLT-R, and WRAT-4 Word Reading, as well as years of education, between participants with and without inconsistencies on the BPI-SF (Table 2). Participants who completed the BPI pain severity items incorrectly had significantly lower RBANs Attention Index Scores (t(68) = 2.09, *P* < 0.05), Delayed Memory Index Scores (t(67) = 2.27, *P* < 0.05), and Total Index Scores (t(67) = 2.30, *P* < 0.05). Individuals who had inconsistent or incorrect BPI responses had lower scores that approached significance on the RBANs Immediate Memory Index (t(68) = 1.67, *P* < 0.10) and Visuoconstruction Index (t(67) = 1.67, *P* < 0.10). In contrast to the other measures of neuropsychological function we examined, the RBANS Language Index did not approach significance between groups. Patients who incorrectly completed the BPI-SF pain severity items had lower HVLT-R Total Recall scores (t(68) = 4.13, *P* = 0.160) and Delayed Recall scores, but these differences were not significantly different. Further, although WRAT-4 Word Reading scores were slightly higher in the group with correct responses, the difference in reading ability was nonsignificant (*P* = 0.167). We found a significant difference in years of education between groups (t(51) = 1.88, *P* < 0.01); the group with correct responses had an average of 2 additional years of education. After adjusting the *P*-values for all tests, the only significant difference between the groups was years of education. The effect sizes found for all these results ranged from 0.06 to 0.96, with most of the neurocognitive results having at least a medium effect (>0.5) size and education having an effect size >0.8. Post-hoc power analyses suggested that these comparisons were significantly underpowered (0.05–0.82) due to small and unequal sample sizes, except for the education comparisons that had power >0.80.

**Table 2:** Comparisons of cognitive function between groups (pain severity items).

Variables	Descriptive statistics					Independent *t* test statistics				Hedge's g
	Mean	SD	Min	Max	SE	Mean Diff	t	df	*P*	
RBANS immediate memory										
Correct BPI	91.93	15.528	49	123	2.094	7.594	1.672	68	0.099	0.482
Incorrect BPI	84.33	15.860	53	109	4.095					
RBANS visuoconstruction										
Correct BPI	83.04	15.387	50	126	2.094	7.304	1.737	67	0.087	0.501
Incorrect BPI	75.73	9.845	60	92	2.542					
RBANS language										
Correct BPI	89.72	14.835	47	120	2.019	2.456	0.539	67	0.591	0.156
Incorrect BPI	87.27	18.203	54	127	4.700					
RBANS attention										
Correct BPI	86.67	16.798	43	122	2.286	10.600	2.093	67	0.040*	0.604
Incorrect BPI	76.07	19.289	53	112	4.980					
RBANS delayed memory										
Correct BPI	94.17	10.095	71	122	1.374	7.233	2.274	67	0.026*	0.656
Incorrect BPI	86.93	13.520	56	100	3.491					
RBANS total										
Correct BPI	85.31	12.466	54	119	1.696	8.381	2.303	67	0.024*	0.665
Incorrect BPI	76.93	12.487	50	94	3.224					
HVLT-R total recall										
Correct BPI	40.33	11.883	19	57	1.320	4.127	1.420	68	0.160	0.665
Incorrect BPI	36.20	10.678	20	58	2.757					
HVLT-R delayed recall										
Correct BPI	37.40	11.883	19	60	1.602	0.686	0.200	67	0.842	0.059
Incorrect BPI	36.71	9.571	20	53	2.558					
WRAT4 reading										
Correct BPI	93.96	12.870	55	126	1.785	3.362	0.913	65	0.364	0.265
Incorrect BPI	90.60	11.338	73	111	2.927					
Education										
Correct BPI	14.02	1.837	11	18	0.287	1.858	2.975	51	0.004*	0.962
Incorrect BPI	12.17	2.125	8	16	0.613					

RBANS and WRAT- 4 scores are age-normed and have a mean of 100 and a standard deviation of 15. HVLT-R are age-normed and have a mean of 50 with a SD of 10. Across measures, lower scores indicate poorer performance.

* *P* < 0.050.

BPI, brief pain inventory; df, degrees freedom; HVLT-R, Hopkins Verbal Learning Test—Revised; RBANS, repeatable battery for the assessment of neuropsychological status; SD, standard deviation; SE, standard error; WRAT4, Wide Range Achievement Test 4.

No significant group mean differences were found in neuropsychological function for patients who completed the body map item inconsistently with the instructions. These data are provided in a supplementary materials, http://links.lww.com/PR9/A246.

## 4. Discussion

Our study compared neurocognitive function and education levels between 2 groups of patients with SCD based on accurate completion of the BPI-SF. We found that 21% of participants gave inconsistent or inaccurate responses on their level of pain in the last 24 hours, and 57.7% of participants did not follow instructions for completing the body image portion of the form. Inaccurate completion of the BPI-SF was significantly associated with education levels in adults with SCD. This finding has important implications in light of previous evidence that patients with SCD have lower academic attainment related to neurological problems^7^ and medical complications that affect school attendance and performance^20^ beginning early in life.

Because very few studies have examined patient error rates on pain forms, it is difficult to say if this high percentage of patients with incorrect responses is unique to patients with SCD. One study of older adults with and without cognitive impairment found much lower rates of error among commonly used pain scales (0%–3.6%).^14^ However, this study did not compare multiple pain items to each other, and we found that conflicting information provided in the pain severity items was the most common error (15.5%). Likewise, the percentage of incorrectly answered BPI-SF by patients with SCD is actually lower in this sample than in a previous study.^25^ The parent study for our secondary analysis required that participants be available for a long panel of neurocognitive tests. This may have biased the sample towards subjects with more resources and education. In the previous study, participants were only asked to complete the BPI and other paper and pencil questionnaires. Further investigation is warranted to determine the true prevalence of completion errors across pain assessment instruments such as the BPI, in this population.

Few studies have examined how education level influences the BPI-SF's ability to measure pain or changes in pain over time, but one study found that the psychometric properties of shorter pain scales were not influenced by education level.^27^ We were unable to find a reading grade level for the BPI in published literature. Based on our analysis, the BPI-SF requires a seventh grade reading level.^40^ Given that the mean education level for both study groups was above US high school education they should have been able to complete it accurately. Consistent with this, word reading level between the groups was not significantly different. These factors suggest that other factors may affect the ability of some patients with SCD to complete the BPI-SF. Although our study did not find significant neurocognitive differences between the 2 groups after controlling for multiple comparisons, effect sizes were generally medium sized, suggesting differences may be detectable with larger samples.

Results from previous studies suggest attention and memory challenges in individuals with SCD,^2,9,48^ which may affect their ability to accurately complete questionnaires. Individuals with SCD are at increased risk of cognitive dysfunction compared to healthy peers due to disease-related CVAs,^47,60,61^ although cognitive dysfunction may also occur in neurologically intact adults.^63^ In addition, SCD is associated with pain and fatigue, which are known to affect attention and memory.^13,49^

A factor that may have influenced results in our study was the increased rate of potentially confusing items on the BPI-SF when compared to other pain rating scales with fewer questions, such as the VAS, NRS, or the Bieri Faces Scale.^27,55^ This difference could contribute to difficulties with accurate completion for participants with SCD who have lower levels of education. Multiple questions on the BPI-SF request information about varying timeframes during which patients experience pain. Item 1 asks about pain today, items 3 and 4 refer to pain in the past 24 hours, item 6 asks about pain at the time of assessment, and items 2 and 5 do not provide patients with a time context to consider when reporting pain symptomology.^15,16^

### 4.1. Limitations

Our study has multiple limitations. Compared with the 113 participants consented in the parent study, only 63% (n = 71) completed the BPI-SF and 79% (n = 88) completed all the baseline neuropsychological testing, bringing into question how well our sample represents the clinic population. In most cases, because the BPI-SF was not prioritized among questionnaires participants were given, many may have run out of time to complete this questionnaire before their study visit was over. Participants likely to have the most difficulty completing self-report measures may have been missed; more than half our sample reported some postsecondary education, compared with a prior study showing only 36.5% of participants with SCD completed postsecondary education.^35^ Moreover, the study team did not directly observe patients with SCD filing in the forms. The study staff assessed forms for completion before participants left the outpatient clinic and encouraged participants to fill in the forms as completely as possible; hence, only 1.4% of the sample had missing items. Observing patients directly in the future may provide additional insights into why participants make mistakes during BPI-SF completion.

Small sample size limited the power of this study. While our education findings were significant, incomplete data limited our ability to detect a significant indepenedent effect of neurocognitive function on error rates. The sample of individuals who made mistakes on the BPI-SF was small and disproportionate to the rest of the sample. In addition, a high number of patients were missing education data (n = 18), 15 of whom were in the group that completed the BPI-SF correctly. Another important consideration is that our study included only participants with SCD, limiting our ability to determine whether responding incorrectly on the BPI-SF is unique to individuals with SCD. While the parent study did include non-SCD controls, only 12 completed the full BPI-SF; therefore, there was no adequate sample to examine this further.

Future studies will benefit from gathering qualitative information to understand how the BPI-SF is interpreted and answered from patients' perspectives. Longitudinal information would be valuable to examine whether neuropsychological functioning and inconsistencies on the BPI-SF in adults with SCD are associated with long-term outcomes such as dementia. Future studies would benefit from obtaining an intelligence quotient. Finally, our sample is restricted to one region in the United States and, therefore, may not capture geographic differences in education and culture that influence interpretation and reporting styles, limiting generalizability of our findings.

## 5. Conclusions

Our study's findings replicated results from a previous study showing mistakes in how participants with SCD completed the BPI-SF^25^ and identified education level as a potential reason for inaccurate responding. At least one in 5 participants in our study showed problems with response accuracy, suggesting they had not interpreted items accurately despite completing the form in a quiet location with as much time as necessary and given the option to discontinue at any time and still receive compensation.

Assessing and treating pain in SCD is challenging, in part due to the subjective nature of pain and the reliance on patients' perception of pain symptoms.^22^ However, accurate and reliable assessment of SCD pain is critical for developing effective interventions^22^ and should rely on well-validated, reliable pain assessment tools and patient interview.^3,22^ Inaccurate reports of pain due to misunderstanding of instructions is a relatively unexplored but significant barrier to the creation and delivery of appropriate pain interventions and clinical care. Despite its limited scope, our study suggests that more careful consideration of education level is warranted when using the BPI-SF to collect pain information from adults with SCD, and potentially other diverse patient populations facing health disparities. Simplifying sentences, shortening sentence length, more clearly specifying timeframes, and perhaps changing the mode of administration (eg, interviewer administered) may lead to more accurate and consistent completion of the form.

## Disclosures

The authors have no conflict of interest to declare.

C.R.J. reports being a co-founder, Chief Executive Officer, and holding equity in Expressive Painimation Co., a company that develops pain assessment technology.

## Supplemental digital content

Supplemental digital content associated with this article can be found online at http://links.lww.com/PR9/A246.

## Supplementary Material

SUPPLEMENTARY MATERIAL
