# Supplementary material for: Cognitive considerations for adults with sickle cell disease completing the brief pain inventory
Source: Pain Rep. 2024 Dec 9;10(1):e1189. doi: 10.1097/PR9.0000000000001189 (PMC11631029; doi:10.1097/PR9.0000000000001189)
Supplement: SUPPLEMENTARY MATERIAL [file painreports-10-e1189-s001.pdf]

Figure 1. Overview of secondary analysis of Brief Pain Inventory-Short Form data.

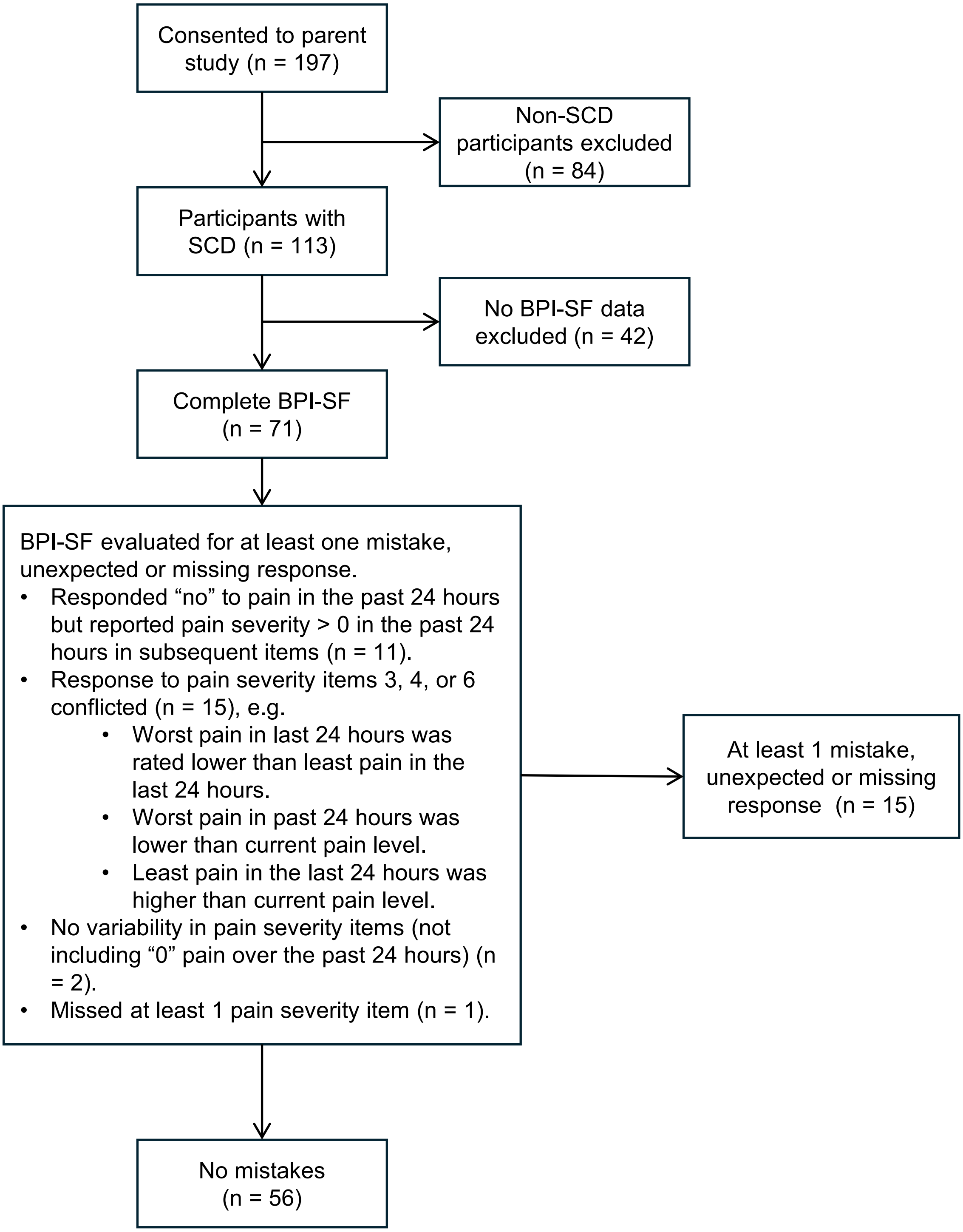

Table 3. Comparisons of Cognitive Function Between Groups (Body Map) (n = 50)

Variables	Descriptive Statistics					Independent T-test Statistics				
	Mean	SD	Min	Max	SE	Mean Diff.	t	df	p-value	Hedge's g
**RBANS Immediate Memory**										
Correct BPI	93.44	14.52	65	109	4.84	3.274	0.538	48	.593	.195
Incorrect BPI	90.17	16.90	49	114	2.64					
**RBANS Visuoconstruction**										
Correct BPI	81.67	10.24	66	100	3.41	-0.103	-0.018	46	.985	-.007
Incorrect BPI	81.77	15.82	60	126	2.53					
**RBANS Language**										
Correct BPI	90.33	10.30	78	104	3.43	2.258	0.372	47	.712	.135
Incorrect BPI	88.08	17.45	51	124	2.76					
**RBANS Attention**										
Correct BPI	87.78	11.13	72	103	3.71	4.553	0.699	47	.488	.254
Incorrect BPI	83.23	18.71	53	122	2.96					
**RBANS Delayed Memory**										
Correct BPI	95.89	8.58	78	108	2.86	3.855	0.810	47	.422	.294
Incorrect BPI	92.20	12.99	56	122	2.05					
**RBANS Total**										
Correct BPI	86.78	9.88	72	102	3.30	3.855	.749	46	.458	.277
Incorrect BPI	82.92	14.63	50	119	2.34					
**HVLT-R Total Recall**										
Correct BPI	41.67	11.60	27	56	3.87	0.917	.248	47	.805	.090
Incorrect BPI	40.75	9.67	20	58	1.53					
**HVLT-R Delayed Recall**										
Correct BPI	39.44	13.30	20	60	4.43	0.675	.160	46	.873	.058
Incorrect BPI	38.77	10.96	20	60	1.76					
**WRAT Reading**										
Correct BPI	91.71	9.74	79	108	3.68	-2.414	-.535	36	.596	-.219
Incorrect BPI	94.13	10.99	73	126	1.97					
**Education**										

Correct BPI	13.63	2.62	11	18	0.93	0.384	.432	35	.669	.169
Incorrect BPI	13.24	2.12	8	17	0.39					

Notes: * p <.050, BPI = Brief Pain Inventory, RBANS = Repeatable Battery for the Assessment of Neuropsychological Status, WRAT4 = Wide Range Achievement Test 4, HVLT-R = Hopkins Verbal Learning Test – Revised, SD = Standard Deviation, SE = Standard Error, df = degrees freedom.

RBANS and WRAT- 4 scores are age-normed and have a mean of 100 and a standard deviation of 15. HVLT-R are age-normed and have a mean of 50 with a SD of 10. Across measures, lower scores indicate poorer performance.
